# What Have We Learned From Family-Based Studies About Spondyloarthritis?

**DOI:** 10.3389/fgene.2021.671306

**Published:** 2021-06-03

**Authors:** Félicie Costantino, Hendrick Mambu Mambueni, Roula Said-Nahal, Henri-Jean Garchon, Maxime Breban

**Affiliations:** ^1^UVSQ, Inserm U1173, Infection et inflammation, Laboratory of Excellence INFLAMEX, Université Paris-Saclay, Montigny-le-Bretonneux, France; ^2^Rheumatology Division, Ambroise Paré Hospital, Boulogne-Billancourt, France; ^3^Genetics Division, Ambroise Paré Hospital, Boulogne-Billancourt, France

**Keywords:** spondyloarthritis, ankylosing spondylitis, genetics, family-based studies, rare variant

## Abstract

Spondyloarthritis (SpA) is a chronic inflammatory disorder with a high familial aggregation, emphasizing the existence of genetic susceptibility factors. In the last decades, family-based studies have contributed to better understand the genetic background of SpA, in particular by showing that the most likely model of transmission is oligogenic with multiplicative effects. Coexistence of different SpA subtypes within families also highlighted the complex interplay between all subtypes. Several whole-genome linkage analyses using sib-pairs or multiplex families were performed in the 1990s to try to identify genetic susceptibility factors besides HLA-B27. Unfortunately, no consistent results were obtained and family-based studies have been progressively set aside in favor of case-control designs. In particular, case-control genome-wide association studies allowed the identification of more than 40 susceptibility regions. However, all these loci explain only a small fraction of disease predisposition. Several hypotheses have been advanced to account for this unexplained heritability, including rare variants involvement, leading to a renewed interest in family-based designs, which are probably more powerful in the detection of such variants. In this review, our purpose is to summarize what has been learned to date regarding SpA genetics from family-based studies, with a special focus on recent identification of rare associated variants through next-generation sequencing studies.

## Introduction

Spondyloarthritis (SpA) is a chronic inflammatory disorder which encompasses several presentations characterized by axial and/or peripheral joint inflammation, often in association with extraarticular inflammation of the eye, skin, or gut (Taurog et al., 2016). One of the striking features of the disease is its high familial aggregation. In this review, our purpose is to summarize what has been learned to date regarding SpA genetics from family-based studies. We also highlight the potential interest of family-based designs for identification of rare associated variants.

## Genetic Epidemiology of SpA

Classical genetic epidemiology approach is based on family studies with three sequential steps addressing the following questions: (1) “does disease cluster in families?,” (2) “is familial clustering related to genetic, environmental, or cultural risk factors?,” and (3) “how is genetic susceptibility inherited?” (King et al., 1984). This approach has been applied to determine and quantify genetic influence in SpA. As for most of the studies in the field of SpA genetics, our knowledge on genetic epidemiology mainly comes from studies restricted to ankylosing spondylitis (AS) phenotype. Restriction to this well-defined prototypical phenotype of SpA, requiring an advanced radiological sacroiliitis, aimed at increasing the genetic homogeneity of the studied cohorts, and thus improving reliability and power of the analyses. However, as discussed later in this review, other subtypes of SpA have been found among family members of patients with AS suggesting shared genetic factors. Taking these cases into considerations may help to provide a comprehensive picture of the genetic factors involved in SpA.

### Do As Cluster in Families?

Familial aggregation studies are commonly the first step in the identification of genetic determinants of disease. Their objective is to determine if the risk to develop a disease is increased if one of the relatives already has this disease. Aggregation studies may be separated into three categories according to their design: population-based, case-control, or family-based studies (Matthews et al., 2008). The most popular approach is to sample independent (i.e., unrelated) patients, called probands, and to obtain their detailed family history of disease. Potential biases include ascertainment bias and/or overreporting of affected cases which might lead to overestimation of familial aggregation (Wickramaratne, 1995; Guo, 1998).

In AS, several family-based approaches have been used to assess recurrence risk ratio (RRR). By compiling data from six studies comprising 4,924 siblings and 466 parents of AS probands, Brown et al. (2000) reported a RRR of 82 in siblings and 79 in parent-child. This estimate was based on a disease prevalence of 0.1% in the general population, lower to that now commonly admitted in European population [0.25%, 95% confidence interval (CI): 0.18–0.33%] which might have led to an overestimation of the risk (Stolwijk et al., 2016). However, similar ratios were obtained in population-based studies in Iceland with a first-degree RRR ranging from 75 to 94 (Thjodleifsson et al., 2007; Geirsson et al., 2010). In contrast, two register-based case-control studies of AS patients in Sweden reported a substantially lower RRR with a sibling risk between 15 and 20 (Sundquist et al., 2008; Morin et al., 2019).

Despite some discrepancies in the magnitude of the risk, familial aggregation studies all demonstrated that AS clusters in families more frequently than expected by chance.

### Is Familial Clustering Related to Genetic, Environmental, or Cultural Risk Factors?

Three general mechanisms, not mutually exclusive, may explain such familial clustering: genetic factors, environmental factors to which related individuals may be exposed together or cultural inheritance of risk factors related to lifestyle. Heritability refers to the proportion of the variation in a trait due to genetic factors (Tenesa and Haley, 2013). Classical study designs to assess it are family-based, mainly twin studies. More recently, SNP-based heritability assessment methods using unrelated cases and controls have been developed (Yang et al., 2010, 2017).

#### Twin Studies

Genetic contributions to a disease may be estimated from twin studies by comparing the phenotypic similarity of monozygotic (MZ) to that of dizygotic (DZ) twins. Indeed, both types of twins are raised together and therefore share a large proportion of environmental exposures and cultural risk factors. However, MZ twins are genetically identical whereas DZ twins share only 50% of their genome. Thus, comparison of the concordance for a trait between MZ and DZ twins allows to estimate its genetic heritability. However, twin design relies on several assumptions which may lead to an overestimation of heritability if not met. In particular, they assume that shared environmental factors are identical in MZ and DZ pairs, which has been questioned (Felson, 2014).

In AS, two twin studies have estimated heritability. The first one was a compilation from four studies including 83 twin pairs (Brown et al., 1997). Concordance rate was higher in the 27 MZ pairs (63%) than in the 56 DZ ones (12.5%). Heritability was estimated to 97% (95% CI: 92–99.2%) based on a disease prevalence of 0.1%. The second one, compiling one Norwegian and two Danish nationwide twin surveys, found lower concordance rates (maybe because of the nationwide population design which minimizes the risk of ascertainment bias) but again with striking differences between MZ (40%) and DZ twins (4.3%) (Pedersen et al., 2008a). In that study, heritability was estimated to 61% but with a broad 95% confidence interval ranging from 0 to 99% because of the small sample size of the study (28 twin pairs only). A similar Danish nationwide study focused on psoriatic arthritis, one of the SpA subtypes, found a lower heritability (34%) (Pedersen et al., 2008b).

#### Single Nucleotide Polymorphism-Based Heritability Studies

With the development of high-throughput genotyping methods, it became possible to estimate the genetic similarity between individuals through the use of whole-genome single nucleotide polymorphism (SNP) array data. Different statistical methods have been developed to test this SNP-based heritability (Yang et al., 2017). A major asset of this approach is the possibility to use unrelated subjects with no risk of confusion due to shared environmental factors and thus to use very large datasets from genome-wide association studies (GWAS). SNP-based heritability assessment detects only the additive effects of causal variants tagged by common SNPs present in the SNP microarray used. Thus, this type of heritability is expected to be lower than twin-based heritability, and the difference between the two methods may reflect the contribution of rare variants or structural variants not included in the microarrays or non-additive effects (Yang et al., 2010).

In AS, SNP-based heritability has been estimated at 60.8% using all the SNPs of Immunochip array and at 27.8% using only the 244 independent association signals significant at genome-wide level (Ellinghaus et al., 2016). This gap suggests that common variants associated with AS are yet to be identified.

More recently, heritability of more than 2,000 traits has been estimated in the UK Biobank (UKBB) cohort using a genome-wide microarray with a better coverage than Immunochip (Abbott and Neale, 2020). In this cohort, AS heritability was estimated at 39.9%. However, this estimate should be interpreted cautiously for several reasons. First, the sample size of only 584 AS patients was too low to yield robust estimate. Moreover, identification of AS cases in this cohort relied on medical records and probably lacked accuracy. Finally, UKBB cohort cannot be considered representative of the United Kingdom population, with a selection bias toward healthier individuals (Fry et al., 2017). This may explain the very low prevalence of AS in this cohort (0.04%) which might inflate the estimated heritability.

Altogether, heritability studies suggest a strong genetic contribution to SpA. The difference observed between the heritability assessments from twins and those from unrelated case–control studies also suggests a potential contribution of rare or structural variants not captured by the Immunochip.

### How Is Genetic Susceptibility Inherited?

Given evidence for genetic influence on disease susceptibility, the next step is to determine how genetic susceptibility is inherited. As for most common diseases, AS does not appear to be inherited as a simple Mendelian dominant or recessive trait. Segregation analyses aimed at determining how susceptibility segregates in families. By comparing observed disease incidence in each relative class with that expected based on a specified model, it is possible to test various genetic hypotheses. Determination of the mode of inheritance of a complex disease is, however, challenging because of numerous potential confounders including genetic heterogeneity, ascertainment bias, and incomplete penetrance (King et al., 1984).

To assess the most likely mode of inheritance of AS, Brown et al. (2000) compared recurrence risk ratios estimated from several genetic models to those observed in different class of relatives of affected subjects according to previously published data. Among the five tested models (“single locus,” “polygenic multiplicative,” “two locus multiplicative,” “HLA, residual polygons,” and “five locus multiplicative”), the best fitting model was a five-locus model with multiplicative interaction between loci. The precise number of genes involved cannot be accurately modeled, and models with three to nine genes were equally consistent with the observed data.

## Phenotypic Family-Based Approaches

Studies of familial cases have also helped to better understand the relationships between SpA subtypes and to refine the clinical description of familial SpA.

### Can We Study Together All SpA Subtypes for Genetic Purpose?

Spondyloarthritis consists of several closely related disorders, including AS, psoriatic arthritis, inflammatory bowel disease-associated SpA, reactive arthritis, and undifferentiated SpA. Although each of these entities is defined by specific characteristics, they share several major epidemiological, clinical, and imaging features, as well as an association with HLA-B27, leading to the unified concept of SpA (Moll et al., 1974). A critical question regarding the concept of SpA concerns the extent to which distinct manifestations belonging to the SpA spectrum depend on identical factors, including genetic predisposition.

Two alternative models have been proposed. The first one hypothesized a genetic heterogeneity, with different combinations of several independent predisposing factors leading to a variety of phenotypic expressions of disease. This assumption was supported by studies suggesting that different disease forms bred true within families (Hochberg et al., 1978; Calin et al., 1984). The alternative model of phenotypic diversity postulated that there is a predominant predisposing component common to most forms of SpA, but different manifestations occur because of the additional influence of minor factors. To test these two models, the French Group for Genetic Research on SpA (GFEGS) has extensively studied SpA manifestations in families with multiple cases of SpA. They showed that distinct SpA subtypes can coexist within families (Said-Nahal et al., 2000) and similar observations were also made in Chinese families (Chou et al., 2005). They also demonstrated that all the articular and extra-articular manifestations belonging to the spectrum of SpA segregated together (Said-Nahal et al., 2001). Finally, they estimated the recurrence risk ratio at 35 for parent/child and 45 for siblings (Dernis et al., 2009). Altogether, these results suggested that SpA subtypes might be considered phenotypic variations of a unique disease and could be studied together in genetic studies.

### Is Disease Severity Genetically Determined in SpA?

Little is known about the genetic control of disease severity in SpA. The concept of severity in itself is not well defined in SpA as severity can include multiple aspects of the disease such as pain, disease activity, impaired physical function, or radiographic structural damage.

By studying AS-affected sib pairs, Calin and Elswood (1989) showed a high familiality of pain and disability indices, as well as radiographic damages. Hamersma et al. (2001) estimated the heritability of disease activity [through Bath Ankylosing Spondylitis Disease Activity Index (BASDAI)] and functional impairment [through Bath Ankylosing Spondylitis Functional Index (BASFI)] in AS. Strong heritability was observed for both indexes (51% for BASDAI and 68% for BASFI) but not for the age at disease onset (18%). High heritability has also been demonstrated for radiographic damages assessed by Bath Ankylosing Spondylitis Radiology Index (Brophy et al., 2004). However, to date, no genetic factors of disease severity has been consistently reported.

### Is There Any Phenotypic Difference Between Familial and Sporadic SpA?

Several studies investigated whether clinical presentation of familial cases differed from that of sporadic cases (i.e., without known affected relative). However, the definition of a “familial” form is not as straightforward as it might appear because the probability of reporting an affected relative is highly dependent on the number of ascertained relatives, i.e., the sibship size.

Factors described as associated with familial disease differed between studies, except for a higher prevalence of HLA-B27 identified in most of them (Almodóvar et al., 2011, 2016; Joshi et al., 2012; Kim et al., 2014). There was also a trend to a milder disease in familial forms (Calin et al., 1993; Almodóvar et al., 2016), which might have been caused by a selection bias among familial cases (the probability to diagnose a patient with mild symptoms might be higher in the presence of a family history of SpA).

Thus, differences of clinical presentation between sporadic and familial cases of SpA seem to be minor.

## Family-Based Approaches for Identification of Genes of Susceptibility to SpA

Historically, family-based designs were the preferred tool used to identify not only genetic factors of susceptibility, especially in Mendelian diseases but also in complex traits with familial aggregation, such as SpA. More recently, there was a renewed interest in family-based approaches for their potential interest in the detection of rare variants.

### Linkage Analyses

Linkage analysis is a genetic method that searches for chromosomal segments that cosegregate with the disease phenotype through families. In SpA, three genome-wide linkage studies using microsatellites were published, two in AS and one in SpA as a whole (Laval et al., 2001; Miceli-Richard et al., 2004; Zhang et al., 2004). In all of them, major histocompatibility complex (MHC) region was highly linked to SpA. However, only two loci besides the MHC reached significance threshold: one on 16q in AS (Laval et al., 2001) and the other on 9q31-34 in SpA (Miceli-Richard et al., 2004).

These findings have been followed up in order to identify more precisely the gene involved in SpA susceptibility in the significantly linked regions. In the first published AS GWAS (Wellcome Trust Case Control Consortium et al., 2007), a suggestive association was found between AS and two SNPs close to the 16q region. This association was further replicated and refined to a region including the gene *TRADD*, involved in NFκB signaling and the regulation of proinflammatory cytokines (Pointon et al., 2010). On the other hand, comprehensive study of the 9q31-34 region, including fine mapping of the whole region and systematic sequencing of a large number of genes in that region led to the identification and replication of a protective haplotype of six SNPs near the *TNFSF15* gene, a Crohn’s disease susceptibility gene (Zinovieva et al., 2009), and of a rare SNP located in *TNFSF8*, a gene which plays a critical role in Th17 cell differentiation (Sun et al., 2010), as significantly associated with SpA (Zinovieva et al., 2008, 2011).

One limitation of linkage studies is that they cannot locate disease-associated loci on a fine scale. To try to circumvent this issue, a more recent linkage analysis used a high-density panel of SNPs and identified a new locus significantly linked with SpA was identified on 13q13 (Costantino et al., 2016). However, despite the higher density of marker, the disease interval could not be restricted to less than 1.4 Mb and further investigations are needed to identify causal variant(s) in this region.

### Family-Based Association Analysis

Familial approach can also be applied to genetic association study. To date, only one family-based association analysis has been published in SpA (Costantino et al., 2017). None of the tested SNPs reached genome-wide significance. However, combined analysis including two independent family-based replication cohorts identified an association close to genome-wide significance between an intronic SNP of *MAPK14* and SpA. Moreover, nominal associations for polymorphisms in several *loci* previously associated with AS through case-control GWAS reinforcing the evidence of shared genetic background between SpA as a whole and AS.

### Rare Variants

To date, case-control genome-wide association studies allowed the identification of more than 40 susceptibility regions outside of the major histocompatibility complex. However, all these loci, including HLA-B27, explain less than 30% of AS heritability (Ellinghaus et al., 2016). Several hypotheses have been advanced to account for this unexplained heritability, including structural variants, gene-gene and gene-environment interactions, and rare variants (Bodmer and Bonilla, 2008; Eichler et al., 2010). However, case-control studies often lack statistical power to detect the latter accurately. As an example, despite a rather large sample size (5,040 patients and 21,133 healthy controls), the only reported case-control study investigating the role of rare variants in AS had a power estimated to 9% for variants with a minor allele frequency (MAF) of 1% and close to zero for variants with MAF of 0.02%, corresponding to the median of the study (Robinson et al., 2016).

Family-based approaches can help in the detection of rare variants potentially involved in SpA. Indeed, variant filtering process is easier in families because of the possibility to analyze the cosegregation of variants with the phenotype under study. Moreover, rare variants are more prone to be population specific and family-based designs are more robust to population stratification than case-control design (Laird and Lange, 2006).

There is an increasing number of studies combining family-based design and next-generation sequencing in SpA (Uddin et al., 2013; Rong et al., 2015; O’Rielly et al., 2016; Feng et al., 2018; Tan et al., 2018; Garshasbi et al., 2020; Liu et al., 2020). They all showed a perfect or at least a high degree of cosegregation of one or several rare variants with SpA in large families with multiple cases of SpA (Table 1). Interestingly, none of these variants or their corresponding genes has been previously identified through GWAS approaches. However, because of the low frequency of these variants, independent replication of associations was challenging and all the studies except one failed to validate the association in an independent cohort (Liu et al., 2020). Experimental validation might be an alternative to genetic replication but should be investigated.

**TABLE 1:** Family-based studies investigating rare variants in SpA.

Study	Design	Ethnicity	Variant (gene)	Discovery sample	Validation sample	Functional consequences
Uddin et al. (2013)	Whole-genome CNV	Caucasian	7 kb duplication (UGT2B17)	1 family (6 AS)	587 AS/584 HC (*p* = 0.09)	No functional data
Rong et al. (2015)	Targeted sequencing	Chinese Han	Arg580Gly (IRS1)	1 family (8 AS)	Not detected in 309 AS and 210 HC	No functional data
O’Rielly et al. (2016)	Whole-exome sequencing	Caucasian	9 bp deletion (SEC16A) and 20 bp deletion (MAMDC4)	1 family (9 AS)	944 AS/1134 HC (*p* = 0.92)	Conformational change (SEC16A) and RNA-mediated decay (MAMDC4)
Tan et al. (2018)	Whole-genome linkage + exome sequencing	Chinese Han	Leu87Val (ANKDD1B)	1 family (5 AS)	Not detected in 500 HC	No functional data
Feng et al. (2018)	Whole-genome linkage + exome sequencing	Chinese Han	Arg213Try (TREML2)	1 family (23 AS)	331 AS/487 HC (*p* = 0.4)	No functional data
Garshasbi et al. (2020)	Whole-exome sequencing	Iranian	Ser2486Gly (RELN)	1 family (7 AS)	Not detected in 50 HC	No functional data
Liu et al. (2020)	Exome sequencing	Chinese Han	Lys132Asn (RNF123)	1 family (2 AS)	994 AS/999 HC (*p* = 0.03)	Decreased ability of monocytes to differentiate into osteoclasts

*CNV, copy number variant; AS, ankylosing spondylitis; HC, healthy controls; *p*, *p* value.*

## Conclusion

Family studies have been critical to demonstrate the genetic background of SpA and to model the transmission of the disease. They also highlighted the connection between all SpA subtypes and reinforced the unified concept of SpA. Recent studies have revealed their potential in the identification of genetic factors involved in SpA susceptibility. Thus, the recent gain of interest in the role of rare variants in complex diseases might lead family-based approaches to return to the front stage.

## Author Contributions

All authors listed have made a substantial, direct and intellectual contribution to the work, and approved it for publication.

## Conflict of Interest

The authors declare that the research was conducted in the absence of any commercial or financial relationships that could be construed as a potential conflict of interest.
